# Is *Osmia bicornis* an adequate regulatory surrogate? Comparing its acute contact sensitivity to *Apis mellifera*

**DOI:** 10.1371/journal.pone.0201081

**Published:** 2019-08-08

**Authors:** Philipp Uhl, Osarobo Awanbor, Robert S. Schulz, Carsten A. Brühl

**Affiliations:** University of Koblenz-Landau, Institute for Environmental Sciences, Landau, Germany; University of Illinois at Urbana-Champaign, UNITED STATES

## Abstract

Bees provide essential ecosystem services and help maintain floral biodiversity. However, there is an ongoing decline of wild and domesticated bee species. Since agricultural pesticide use is a key driver of this process, there is a need for a protective risk assessment. To achieve a more protective registration process, two bee species, *Osmia bicornis*/*Osmia cornuta* and *Bombus terrestris*, were proposed by the European Food Safety Authority as additional test surrogates to the honey bee *Apis mellifera*. We investigated the acute toxicity (median lethal dose, LD50) of multiple commercial insecticide formulations towards the red mason bee (*O. bicornis*) and compared these values to honey bee regulatory endpoints. In two thirds of all cases, *O. bicornis* was less sensitive than the honey bee. By applying an assessment factor of 10 on the honey bee endpoint, a protective level was achieved for 87% (13 out 15) of all evaluated products. Our results show that *O. bicornis* is rarely an adequate additional surrogate species for lower tier risk assessment since it is less sensitive than the honey bee for the majority of investigated products. Given the currently limited database on bee species sensitivity, the honey bee seems sufficiently protective in acute scenarios as long as a reasonable assessment factor is applied. However, additional surrogate species can still be relevant for ecologically meaningful higher tier studies.

## Introduction

Bees are important pollinators of wild and cultivated flora, which makes them essential providers of ecosystem services and maintainers of floral biodiversity [1, 2]. Aside from the honey bee, *Apis mellifera*, there are other managed bees along with a broad spectrum of wild bee species that contribute substantially to plant pollination [3]. However, there is an ongoing trend of wild bee species decreasing in abundance and diversity all over the world [4]. Furthermore, honey bee hive numbers are also substantially decreasing in North America and many European countries [5]. Among various environmental factors, e.g. habitat loss and fragmentation, parasites, agricultural pesticide use has been identified as one of the key drivers of bee decline [6]. The ecological challenge of flying insect decline in general seems to have been underestimated and consequently disregarded in the past. As a recent study by Hallmann et al. (2017) shows, there has been a severe 75% decline in flying insect biomass in several German natural reserves over roughly the last three decades [7].

In the European agricultural landscape, bees can be exposed to a variety of pesticides that target all major pests: herbicides, fungicides, insecticides [8, 9]. They are not only contaminated during foraging on crops but also from visitations of field-adjacent wild flowers [10]. Bees can be exposed to pesticides by direct overspray as well as oral uptake of and contact with nectar and pollen while foraging [11, 12]. They can also be fed contaminated pollen and nectar as larvae. Furthermore, there is potential uptake of soil residues by adults and larvae of soil-nesting species [11–13]. Moreover, consumption of non-nectar fluids such as puddle water, guttation droplets or extrafloral nectar may also lead to contamination [11, 12, 14, 15]. Consequently, bee species are exposed to pesticides through various environmental matrices throughout their lifespan [11, 12].

To prevent adverse impacts of pesticide applications on bee populations, toxic effects of these substances on bee species need to be understood. However, the majority of toxicity testing in laboratory and field setups has been performed using the honey bee, a bred livestock species, whereas all other bee species are far less well-understood in their sensitivity [13].

Furthermore, the honey bee is the only pollinator species that is tested for its reaction towards pesticides in the current risk assessment scheme according to Regulation (EC) 1107/2009 [16]. However, other bee species (i.e. bumble bees, solitary bees) may show quite different responses to pesticide exposure due to differences in physiology and ecology [17]. To account for these significant differences and collect information regarding the sensitivity of bumble bees and solitary bees, the European Food Safety Authority (EFSA) proposed the inclusion of additional surrogate species into EU pesticide risk assessment: The buff-tailed bumble bee, *Bombus terrestris*, and an *Osmia* species (the red mason bee, *Osmia bicornis* or the European orchard bee, *Osmia cornuta*) [18]. However, there has been reasonable doubt that these two species are adequate to provide additional safety in lower tier risk assessment. Uhl et al. (2016) tested five European bee species in acute contact exposure scenarios with a formulated insecticide product (PERFEKTHION^®^) containing dimethoate, which is often used as a toxic standard in regulatory testing [19]. They found that *B. terrestris* and *O. bicornis* were the least sensitive species when compared to a dataset of their own results and collected literature data. Another study by Heard et al. (2017) compared the acute oral sensitivity of the honey bee towards several pesticides (active ingredients) to *B. terrestris* and *O. bicornis* [20]. They found contrasting sensitivity ratios depending on substance since both non-*Apis* bee species were sometimes more, and sometimes less, sensitive. *Bombus terrestris* was generally less sensitive than the honey bee in acute toxicity studies that were compiled by Arena & Sgolastra (2014) [17]. They could not collect *O. bicornis*/*O. cornuta* data, but other *Osmia* species (*O. cornifrons*, *O. lignaria*) were usually also more resistant to toxicant stress than *A. mellifera*. Moreover, EFSA (2013) proposed an assessment factor of 10 to account for interspecific differences when testing only honey bees [18]. This approach proved to be protective in 95% of cases in the meta-analysis by Arena & Sgolastra (2014) [17]. It is unclear, however, if this factor would be protective for the proposed test species due to the slim database of their sensitivity [19, 20].

There is a need to assess the suitability of the new test species that EFSA proposed. Only sensitive species will reduce uncertainty in lower tier risk assessment. However, with the current database, it is not possible to properly evaluate whether the proposed species are adequate. Therefore, we tested one of these proposed surrogate species, *O. bicornis*, with commercial formulations of multiple common insecticides. We performed acute contact toxicity laboratory tests to derive 48h contact median lethal doses (LD50s). We wanted to assess the acute toxic potency of several insecticides from various classes on *O. bicornis*. Furthermore, our goal was to compare those toxicity endpoints to honey bee data from pesticide regulation that are used to assess their safety regarding bees. This enabled us to evaluate whether *O. bicornis* is usually more sensitive than the honey bee, which would make it a suitable additional surrogate species for lower tier risk assessment. Additionally, we examined if an assessment factor of 10 is protective when comparing honey bee to *O. bicornis* sensitivity.

## Materials and methods

### Insecticides

The majority of tested insecticides were chosen with respect to the application frequency of their commercial products in apple, grapes and winter oilseed rape (Table 1) which represent three main cultivation types in Germany [21]. Additionally, formulations of four insecticides that are not frequently applied were included because of the following reasons: Imidacloprid exposure has been implicated as a major factor in bee decline [13]. Dimethoate is often used as a toxic reference in bee ecotoxicity studies. Chlorpyrifos was chosen for inclusion as a second organophosphate insecticide in addition to dimethoate. Furthermore, flupyradifurone is a relatively new insecticide with low acute toxicity towards honey bees for which registration has been applied for use in multiple EU countries [22]. Insecticides were assigned to pesticide classes according to the Compendium of Pesticide Common Names [23]. Representative formulated products that contain those pesticides as active ingredients (a.i.) were chosen for testing (Table 1). Most of these formulations are, or were, registered in Germany in recent years aside from Pyrinex^®^ (a.i. chlorpyrifos) and Sivanto^®^ SL 200 G (a.i. flupyradifurone). To ease readability, only active ingredient instead of formulated product names are used hereafter.

**Table 1:** Tested insecticides and their usage in German agriculture.

Insecticide (a.i.)	Class	Usage share of a.i. [%] per culture (2015/2016)			Tested product
		apple	grapes	winter oilseed rape	
alpha-cypermethrin	pyrethroid	/	/	16.8 / 16.1	FASTAC^®^ SC
beta-cyfluthrin	pyrethorid	/	/	12.1 / 13.3	Bulldock^®^
deltamethrin	pyrethorid	/	/	3.4 /	Decis^®^ Forte
etofenprox	pyrethroid	/	/	12.4 / 18.5	Trebon^®^ 30 EC
lambda-cyhalothrin	pyrethroid	/	/ 3.3	19.5 / 24.6	Karate^®^ Zeon
zeta-cypermethrin	pyrethorid	/	/	2.8 /4.5	Fury^®^ 10 EW
acetamiprid	neonicotinoid	5.2 /8.4	/	2.0 /	Mospilan^®^ SG
imidacloprid	neonicotinoid	/	/ 3.0	/	Confidor^®^ WG 70
thiacloprid	neonicotinoid	12.5 / 10.2	/	16.1 / 6.9	Calypso^®^
dimethoate	organophosphate	/	/	/	PERFEKTHION^®^
chlorpyrifos	organophosphate	/	/	/	Pyrinex^®^
chlorantraniliprole	pyridylpyrazole	23.7 / 26.9	/	/	Coragen^®^
flupyradifurone	unclassified	/	/	/	Sivanto^®^ SL 200 G
indoxacarb	oxadiazine	3.8 / 3.3	44.3 / 34.6	2.3 / 2.9	AVAUNT^®^ 150 EC
pirimicarb	carbamate	19.5 / 15.0	/	/	Pirimor^®^
spinosad	spinosyn	/	/ 27.7	/	SpinTor^®^

The usage share signifies the prominence of a certain compound with regard to all pesticide applications. It is based on the standardised treatment index (STI) which is defined as the number of pesticide applications in a crop in relation to the application rate and cultivated area [21, 24]. Data from Julius Kühn-Institut (2018) [21].

### Experimental procedure

The red mason bee, *Osmia bicornis* (Linneaus, 1758), was used as test species. Bees were ordered as uneclosed adults in cocoons (WAB-Mauerbienenzucht, Konstanz, Germany), received at the end of February 2017 and stored dry at 4°C until experimental preparation started.

Acute, contact toxicity of 16 insecticide formulation towards *O. bicornis* females was investigated (see S1 Table for a timeline of the experiments). To that end, a protocol for solitary bee acute contact toxicity testing from the International Commission on Plant-Pollinator Relationships (ICPPR) was followed or partly adapted [25]. This protocol is a precursor of a standardised testing guideline. Prior to the experiments, bee cocoons were taken from the refrigerator and placed in an environmental chamber at test conditions to let females hatch. Male bees were also collected after hatching to prevent mating with females and used for range finding tests. Female bees’ eclosion time was usually between five to seven days. After eclosion, females were again stored at 4°C until one day before application to reduce stress until enough individuals for a test were available. At this date, they were transferred in to the environmental chamber in test cages (1 L plastic boxes sealed with a perforated lid) and fed *ad libitum* with sucrose solution 50% (w/w) through 2 mL plastic syringes to acclimatise overnight. Twenty bees were assigned to each treatment (usually 5 per cage, n = 4). See the raw data for details on individual study setups [26]. Environmental conditions were set to 16:8h day/night cycle, 60% relative humidity and 21°C. In the summer of 2017 there was a malfunction of the environmental chamber which caused the light to stay on throughout the whole day. Two test runs were therefore conducted with constant lighting (dimethoate, indoxacarb). Since control mortality was below the quality criterium of 10% in those runs, they were evaluated as valid, nonetheless. Anaesthetisation of bees was necessary before the transfer to test cages. To achieve a calm state, bees were chilled at 4°C. During this process they were also weighed. Bees were anaesthetised a second time before treatment application which was performed in a petri dish. In cases where the ambient temperature was too high to keep bees calm after chilling, petri dishes were put on ice for additional cooling. Moribund bees were rejected and replaced with healthy bees prior to the test start.

Treatment solutions were prepared as follows: a control of deionised water containing 0.5% (v/v) wetting agent (Triton^™^ X-100, Sigma-Aldrich) and at least five treatment solutions of the respective insecticide. Concentrations and number of insecticide treatments were determined after conducting range finding tests with male bees before the main test. Results of these pretests were extrapolated to females using the weight difference of both sexes. Insecticide solutions were prepared by diluting the respective concentration in deionised water containing 0.5% wetting agent. In the first tests, bees were applied with 2 *μ*L treatment solution on the dorsal side of the thorax between the neck and wing base using a Hamilton micro syringe (Hamilton Bonaduz AG). Due to easier handling, an Eppendorf Multipette^®^ plus (Eppendorf AG) was used later for most of the tests. In three tests (chlorantraniliprole, flupyradifurone, pirimicarb), the applied volume had to be increased to 4 *μ*L to dilute high doses. See the raw data for details [26]. After ten to 15 min the treatment solution was fully absorbed and a paper tissue was inserted into test cages to provide a hiding place. Following the application bees were returned to the environmental chamber and fed 50% sucrose solution *ad libitum*. Mortality was assessed after 24, 48, 72 and 96h. For dimethoate, a second test run was performed as part of an ICPPR ring test. Control mortality after 48h was ≤10% in all experiments except for flupyradifurone and chlorantraniliprole (both 15%). Those two cases were evaluated and are considered valid since in the ICCPR test protocol it is discussed that control mortality thresholds might be increased to 15 or 20% in the long run.

### Data analysis

Median acute lethal dose values (contact 48h LD50) were calculated for all tested insecticidal products by fitting a dose-response model to the data. Raw data are available through an online repository [26]. Models were chosen by visual data inspection and using Akaike information criterion (AIC). Furthermore, it was ensured that appropriate models were used for tests with control mortality (no fixed lower limit). Where multiple LD50 values were available, a geometric mean LD50 was computed. Weight-normalised LD50 values were further calculated by dividing LD50 values by mean fresh weight of all bees in a respective test. All statistical analyses were conducted with R 3.4.4 [27]. We used the “drc” package [28] for dose-response modeling (version 3.0-1). Honey bee contact 48h LD50 values were gathered by screening regulatory documents (EC review, report, EFSA conclusion, rapporteur member state draft/renewal assessment reports). Furthermore, we contacted national and European authorities, manufacturers and EFSA to collect data and verify them. For a detailed account of the data collection process and various data sources please see S1 Appendix and S2 and S3 Tables. To compare *A. mellifera* and *O. bicornis* endpoints, sensitivity ratios (R = LD50_*A. mellifera*_ / LD50_*O.bicornis*_) were calculated according to Arena & Sgolastra (2014) for all tested insecticides [17]. Honey bee endpoints were not available as weight-normalised values. Therefore, sensitivity of both species could only be compared without taking the weight of test individuals into account.

## Results

Sensitivity of *O. bicornis* towards all tested insecticides varied considerably (Table 2, S1–S17 Figs). The maximum LD50 value of pirimicarb was 3679 times higher than the minimum LD50 of imidacloprid. The median LD50 value of all pesticides was 1.21 *μ*g a.i./bee. About 69% of substances had LD50 values below 2 *μ*g a.i./bee whereas 38% had LD50s under 0.2 *μ*g a.i./bee. Bee mean fresh-weight differed across all tests (range 77.7 to 112.7 mg, mean of all tests 91.6 mg). The indoxacarb test that included the heaviest bees shows a 23% deviation and the thiacloprid test with the least heavy bees a 15% deviation from mean weight. Such variations subsequently also occur in weight-normalised LD50 values.

**Table 2:** Comparison of *O. bicornis* acute contact toxicity with honey bee regulatory endpoints.

Pesticide	*O. bicornis*					*A. mellifera*	R
	LD50	95% CI	Fresh weight	Weight-normalised LD50	95% CI	LD50	
	[*μ*g a.i./bee]		[mg]	[*μ*g a.i./g bee]		[*μ*g a.i./bee]	
zeta-cypermethrin	0.13	0.09 − 0.17	100.8	1.31	0.93 − 1.69	0.002	<0.1
spinosad	2.06	1.61 − 2.51	80.0	25.73	20.13 − 31.33	0.05	<0.1
indoxacarb	1.26	0.90 − 1.63	112.7	11.21	7.94 − 14.48	0.08	0.1
dimethoate	1.32	1.14 − 1.49	99.9	13.20	11.44 − 14.89	0.111	0.1
pirimicarb	115.07	95.96 − 134.18	85.6	1343.61	1120.47 − 1566.74	36.1	0.3
alpha-cypermethrin	0.24	0.16 − 0.33	85.9	2.84	1.89 − 3.80	0.09	0.4
lambda-cyhalothrin	0.14	0.10 − 0.17	93.5	1.45	1.06 − 1.85	0.055	0.4
deltamethrin	0.06	0.04 − 0.07	100.1	0.57	0.43 − 0.71	0.029	0.5
chlorpyrifos	4.19	2.91 − 5.46	92.9	45.07	31.37 − 58.78	3.19	0.8
beta-cyfluthrin	0.04	0.02 − 0.05	100.4	0.35	0.20 − 0.50	0.032	0.9
flupyradifurone	10.59	6.06 − 15.11	83.0	127.52	72.96 − 182.08	17.1	1.6
acetamiprid	1.72	0.85 − 2.59	95.0	18.10	8.96 − 27.23	9.26	5.4
imidacloprid	0.03	0.03 − 0.04	94.6	0.33	0.27 − 0.39	0.245	7.8
chlorantraniliprole	5.92	4.26 − 7.57	79.0	74.91	53.94 − 95.87	>100	16.9
thiacloprid	1.16	0.74 − 1.58	77.7	14.91	9.50 − 20.31	20.8	18.0
etofenprox	0.18	0.14 − 0.22	84.9	2.09	1.63 − 2.55	NA	NA

Insecticides are ordered by sensitivity ratio.

**Fig 1:**
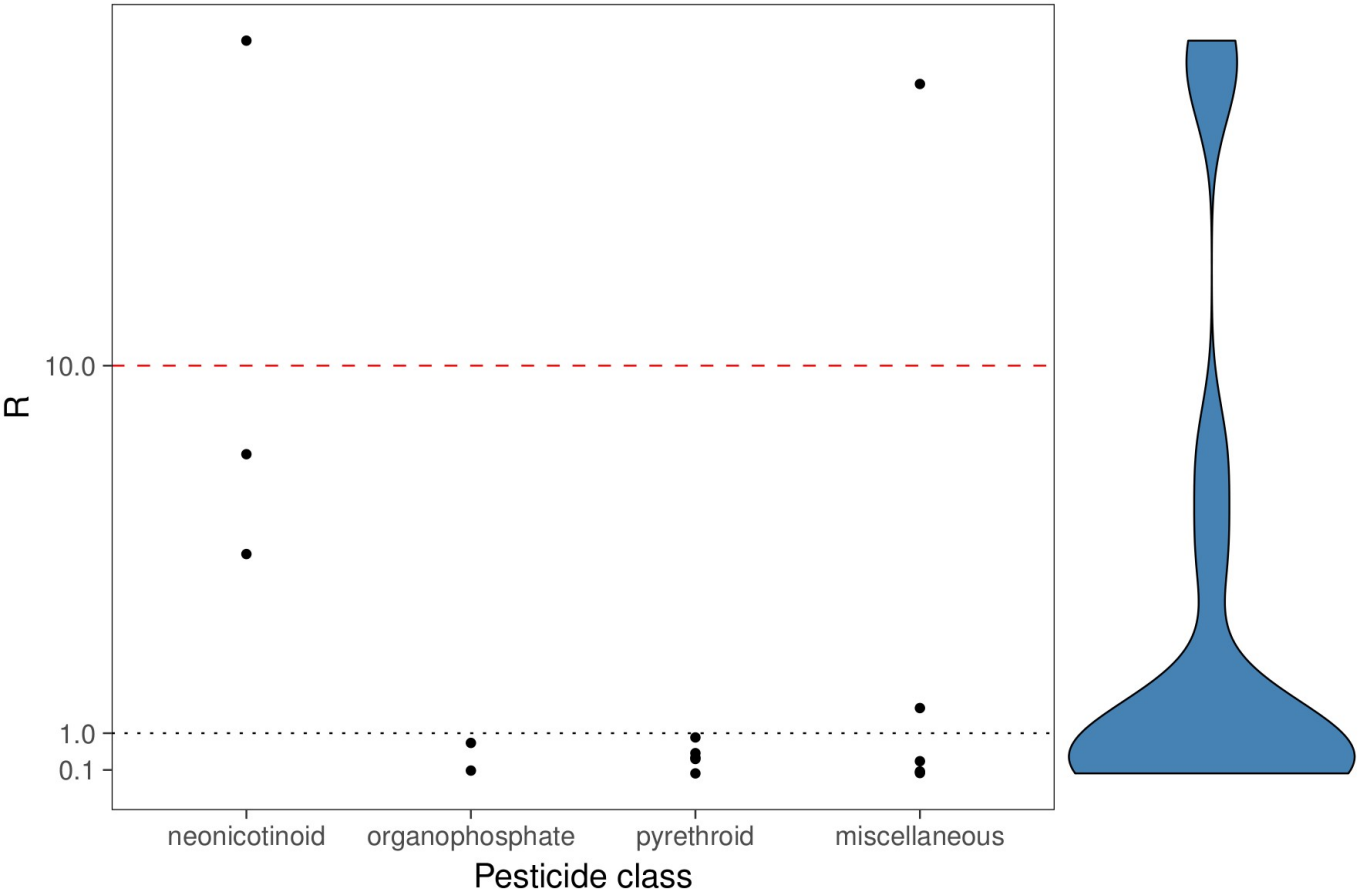
Sensitivity ratio (R) of all tested insecticides grouped by insecticide class. The dotted, grey line signifies equal sensitivity of *O. bicornis* and *A. mellifera*. The dashed, red line indicates the insecticides whose toxicity towards *O. bicornis* would be covered when dividing the honey bee endpoint by an assessment factor of 10. The violin plot on the right shows the distribution of data points.

In two thirds of all cases, *O. bicornis* was less sensitive than the honey bee (15 out of 16 insecticides could be evaluated; no regulatory honey bee data are available for etofenprox product). When dividing the respective honey bee endpoint by an assessment factor of 10, it was lower than the *O. bicornis* endpoint for 87% of all tested substances (Table 2). The two remaining insecticides where *O. bicornis* would still be more sensitive are formulations of chlorantraniliprole and thiacloprid. When analysing sensitivity ratios by insecticide class, it was shown that for organophosphates and pyrethroids values are all below one, i.e. *O. bicornis* was less sensitive than the honey bee (Fig 1). In the case of the three tested neonicotinoids, *O. bicornis* was always more sensitive.

## Discussion

In our study, we assessed the acute contact toxicity of several insecticides from several classes towards *O. bicornis*. Our goal was to compare these data to honey bee endpoints obtained from the pesticide registration process to infer on the suitability of *O. bicornis* as an additional regulatory surrogate species. Furthermore, we wanted to infer if applying an assessment factor of 10 on honey bee LD50 values would be protective for *O. bicornis*.

Acute sensitivity of *O. bicornis* varied substantially between pesticides, which was expected given that the available honey bee endpoints also vary considerably (Table 2). Mean *O. bicornis* female weight also fluctuated between tests, which might have slightly affected their measured sensitivity. However, this effect was not big enough to affect the ranking of insecticides when ordered by acute toxicity. Therefore, these LD50 values are still valid for the comparison with regulatory honey bee values. Since bee individual weight is one factor that influences sensitivity towards pesticides [19], calculating toxicity on a per weight basis leads to more precise and comparable results. Consequently, acute toxicity endpoints should generally also be reported in a weight-normalised format (see Table 2).

To create a more protective environmental risk assessment for bees, EFSA (2013) proposed the inclusion of two additional bee species as surrogates (*B. terrestris*, *O. bicornis*/*O. cornuta*) [18]. These species should accompany the current sole test species, the honey bee. However, in acute toxicity testing, the addition of new species is only reasonable if they are generally more sensitive than the test species already in place. For two thirds of the insecticides we tested, *O. bicornis* was indeed less sensitive than the honey bee (Table 2). This trend is in agreement with the findings of Uhl et al. (2016) who performed acute contact toxicity tests with five bee species and combined their dataset with LD50 values taken from literature [19]. They found that two proposed test species, *O. bicornis* and *B. terrestris*, were less sensitive towards dimethoate than several bee species, including the honey bee. Heard et al. (2017) conducted acute to chronic oral tests (up to 240h) with *B. terrestris* and *O. bicornis* and five organic pesticides, cadmium and arsenic [20]. Their results were inconclusive as to whether the proposed additional test species or the honey bee was acutely more sensitive. If only acute endpoints are considered, *O. bicornis* was more sensitive for two out of six substances that could be evaluated (48h LD50; clothianidin, tau-fluvalinate).

When evaluating this combined information, it becomes evident that *O. bicornis* (and possibly *B. terrestris*) is seldomly an adequate supplementary surrogate species for acute testing of pesticides, since its inclusion would not provide additional safety for the risk assessment process for most pesticides. There is insufficient data to evaluate *O. cornuta*. As postulated by Uhl et al. (2016), test species should be chosen according to their sensitivity in acute effect studies [19]. However, the proposed test species were selected because they are bred for commercial pollination, can be obtained easily in large numbers and cope well under laboratory conditions. While those criteria are important for conducting laboratory experiments in general, they should not be decisive for the selection of surrogate species. The honey bee may be a better choice in acute contact toxicity tests since the not fully matured cuticle of young workers makes it more susceptible towards pesticides compared to solitary bees [29, 30]. Furthermore, there are differences in the immune response of young adults. In honey bees, the individual detoxification capacity is relatively low after hatching and increases from thereon as they age [31, 32]. However, antioxidant enzyme levels already rise in *O. bicornis* adults before eclosion, which is another explanation for their lower sensitivity towards pesticides compared to honey bees at least at this life stage [30].

We could show for 87% of the tested insecticides that dividing the honey bee endpoint by an assessment factor of 10 is sufficient to cover *O. bicornis*’ sensitivity (Fig 1). This assessment factor was found to be protective in 95% of all cases that were analysed in the meta-analysis of Arena & Sgolastra (2014) [17]. After testing multiple bee species with dimethoate, Uhl et al. (2016) reaffirmed this conclusion using a species sensitivity distribution (SSD) approach [19]. Moreover, Heard et al. (2017) state that the honey bee is also an adequate surrogate species for acute oral testing as long as a reasonable assessment factor is applied [20]. A factor of 10 would also have been protective for *O. bicornis* in their study of acute oral toxicity. However, they note that there are exceptions for some substances, e.g. neonicotinoids. Arena & Sgolastra (2014) already mentioned that for this class, wild bee species showed equal or higher sensitivity than the honey bee [17]. This trend is also visible in our data: *O. bicornis* was more sensitive towards all three tested neonicotinoids (acetamiprid, imidacloprid, thiacloprid) than the honey bee (maximum 18 times; Fig 1).

Consequently, the honey bee is a sufficient surrogate species to assess acute toxicity of most pesticides. In some cases (e.g. neonicotinoids) it might be necessary to increase the assessment factor to >10 to achieve a proper level of safety in lower tier risk assessment. To distinguish these substance classes that are relatively more harmful to wild bees than honey bees, a comprehensive ecotoxicological database should be established that includes a representative amount of species and pesticides. Such a database would be helpful for policy-makers to determine protective assessment factors and also for choosing suitable additional test species, if necessary. Moreover, regulatory reporting standards should be improved. Our search for honey bee endpoints from the registration process proved to be complicated. We partly received contrasting information from several sources. A solution for this problem would be the creation of a transparent and publicly available database of regulatory data. Those data could be then complemented by non-regulatory study results to further not only the open science idea but also establish a more transparent regulation process.

Despite only rarely providing additional safety for lower tier risk assessment it should be noted that the proposed test species may be more valuable surrogates in more realistic experimental setups in higher tier risk assessment. Due to their ecological differences to the honey bee, populations of *O. bicornis*/*O. cornuta* and *B. terrestris* may react quite differently in (semi-)field studies. Such divergent effects have been shown in a Swedish field study where clothianidin/beta-cyfluthrin treatment of oilseed rape had no detectable adverse effects on honey bee colonies, yet substantial impact on *O. bicornis*’ and *B. terrestris*’ population development [33]. Therefore, they are good representatives to measure ecological impact of pesticides on solitary and bumble bees in large field studies such as Peters et al. (2016) and Sterk et al. (2016) [34, 35].

## Conclusion

For the majority of substances we tested, the honey bee was more sensitive than *O. bicornis*. We, therefore, agree with Heard et al. (2017) that *A. mellifera* is a sufficient proxy for other bee species in laboratory acute mortality testing as long as an appropriate assessment factor is applied [20]. Dividing the honey bee endpoint by a factor of 10 proved to be protective for *O. bicornis* for 87% of all tested insecticides. There might be exceptions (e.g. neonicotinoids) where this assessment factor needs to be increased. In our dataset, *O. bicornis* was at most 18 times more sensitive than the honey bee. However, an assessment factor should be carefully chosen after consulting a comprehensive bee acute toxicity database. Furthermore, it is still necessary to investigate less well-known issues such as effects of pesticides mixtures [36, 37], prolonged pesticides exposure [20] or effects of pesticide adjuvants [38] on wild and managed bee species.

Our study provides further evidence that *O. bicornis* is rarely an adequate surrogate species to improve lower tier risk assessment [19]. Unnecessary acute studies with non-sensitive species should not be conducted. Only sensitive species should be chosen as additional surrogates to reduce overall uncertainty. However, we agree that the proposed test species can be appropriate in higher tier risk assessment. In complex field settings, ecological differences between the honey bee, bumble bees and solitary bees are more relevant [11, 12, 33]. Therefore, such realistic experiments are better suited to evaluate the overall impact of pesticides on bee species. Consequently, we believe that (semi-)field data should be relied upon to a greater extent than laboratory results in bee risk assessment.

## Supporting information

S1 Appendix Data collection of regulatory honey bee endpoints. (PDF)

S2 Appendix ICPPR solitary bee acute contact toxicity test protocol. (PDF)

S1 Table Overview of all tested insecticides and test dates. For a detailed account of raw data from all tests see Uhl et al. (2018) [26].
(PDF)

S2 Table Data sources of honey bee acute endpoints for all tested insecticides. (PDF)

S3 Table Different organisations that aided with data collection and contact at the respective institutions. (PDF)

S1 Fig Dose-response curve from *O. bicornis* 48h contact toxicity test with beta-cyfluthrin. (PDF)

S2 Fig Dose-response curve from *O. bicornis* 48h contact toxicity test with deltamethrin. (PDF)

S3 Fig Dose-response curve from *O. bicornis* 48h contact toxicity test with zeta-cypermethrin. (PDF)

S4 Fig Dose-response curve from *O. bicornis* 48h contact toxicity test with dimethoate. (PDF)

S5 Fig Dose-response curve from *O. bicornis* 48h contact toxicity test with dimethoate. (PDF)

S6 Fig Dose-response curve from *O. bicornis* 48h contact toxicity test with indoxacarb. (PDF)

S7 Fig Dose-response curve from *O. bicornis* 48h contact toxicity test with acetamiprid. (PDF)

S8 Fig Dose-response curve from *O. bicornis* 48h contact toxicity test with chlorpyrifos. (PDF)

S9 Fig Dose-response curve from *O. bicornis* 48h contact toxicity test with alpha-cypermethrin. (PDF)

S10 Fig Dose-response curve from *O. bicornis* 48h contact toxicity test with chlorantraniliprole. (PDF)

S11 Fig Dose-response curve from *O. bicornis* 48h contact toxicity test with etofenprox. (PDF)

S12 Fig Dose-response curve from *O. bicornis* 48h contact toxicity test with flupyradifurone. (PDF)

S13 Fig Dose-response curve from *O. bicornis* 48h contact toxicity test with imidacloprid. (PDF)

S14 Fig Dose-response curve from *O. bicornis* 48h contact toxicity test with lambda-cyhalothrin. (PDF)

S15 Fig Dose-response curve from *O. bicornis* 48h contact toxicity test with pirimicarb. (PDF)

S16 Fig Dose-response curve from *O. bicornis* 48h contact toxicity test with spinosad. (PDF)

S17 Fig Dose-response curve from *O. bicornis* 48h contact toxicity test with thiacloprid. (PDF)
