# Supplementary material for: Is Osmia bicornis an adequate regulatory surrogate? Comparing its acute contact sensitivity to Apis mellifera
Source: PLoS One. 2019 Aug 8;14(8):e0201081. doi: 10.1371/journal.pone.0201081 (PMC6687126; doi:10.1371/journal.pone.0201081)
Supplement: S1 Appendix — (PDF) [file pone.0201081.s001.pdf]

S1 Data collection of regulatory honey bee endpoints

Honey bee acute contact LD50 values were obtained from registration documents which included EC review reports EFSA conclusion and EC rapporteur state draft/renewal assessment reports (DAR/RAR). If data were avaiblable from DAR/RAR they were taken from these documents. If otherwise EC review reports and EFSA conclusion were consulted (Table S2). In two cases, values could not be procured from publicly available documentation but were provided in a personal communication from the German Environment Agency (UBA). Within these regulatory documents, formulated products are often called by code names. We matched those code names to the commercial names of the products to the best of our knowledge. In cases where honey bee endpoints were only available on a per formulated product basis, those values were converted to per active ingredient basis using the density at 20℃ from safety data sheets. To avoid possible mistakes and validate the collected data, all endpoints were counterchecked with information that was provided by German national authorities, EFSA and manufacturers (Table S3).
