# Supplementary material for: Is Osmia bicornis an adequate regulatory surrogate? Comparing its acute contact sensitivity to Apis mellifera
Source: PLoS One. 2019 Aug 8;14(8):e0201081. doi: 10.1371/journal.pone.0201081 (PMC6687126; doi:10.1371/journal.pone.0201081)
Supplement: S2 Appendix — (PDF) [file pone.0201081.s002.pdf]

S2 ICPPR solitary bee acute contact toxicity test protocol

Solitary bee, Acute Contact Toxicity Test
Version: March 2016

ICPPR workgroup non-Apis bees
Eds. Ivo Roessink, Jozef J.M. van der Steen, Nicole Hanewald

INTRODUCTION

1. This test guideline is a laboratory test method, designed to assess the acute contact toxicity of pesticides and other chemicals to adult solitary bees. It is based principally on the OECD guidelines for the testing of chemicals 214 [1] and Methods to determine the acute oral and contact LD_{50} of pesticides for bumble bees (*Bombus terrestris* L.)[2] and results of the discussions regarding ring testing solitary bees during the meeting of the ICPPR non-Apis testing working group, March 6th, 2014 in Niefern, Germany, February 19th, 2015 in Limburgerhof, Germany and February 29th, 2016 in Braunschweig, Germany.

INITIAL CONSIDERATIONS

2. In the assessment and evaluation of toxic characteristics of substances, determination of acute contact toxicity in solitary bees may be required, e.g. when exposure of these bees to a given chemical is likely. The acute contact toxicity test is carried out to determine the inherent toxicity of pesticides and other chemicals. The results of this test should be used to define the need for further evaluation. In particular, this method can be used in step-wise programmes for evaluating the hazards of pesticides to bees, based on sequential progression from laboratory toxicity tests to semi-field and field experiments [1]. Pesticides can be tested as either active ingredients (a.i.) or as formulated products.
3. The effect of pesticides on solitary bees depends on the body size of the test subject. As solitary bee workers between different species, within one 'colony' of one species and between 'colonies' can have significantly different sizes and related weights, they have a different surface to volume ratio. This affects the susceptibility of these individuals to plant protection products. Smaller bees have a greater surface to volume ratio and have less weight [3][4]. For practical reasons not the surface to volume ratio of solitary bees is assessed but instead the bees are weighed. In this way the LD_{50} can be calculated as µg PPP bee^{-1} and µg PPP gram bee^{-1} which will make the evaluation of the LD_{50} for non-apis bees more consistent.
   To avoid great variation in susceptibility in one test, solitary bees of an average size / weight must be selected and tested.
4. The method is tested on *Osmia* sp. and may be adjusted for other solitary bees.
5. Definitions used are given in the Annex.

PRINCIPLE OF THE TEST

6. Adult female solitary bees are exposed to a range of doses of the test substance dissolved in appropriate carrier, by direct application to the dorsal side of thorax (droplets). The test duration is at max 96 h. If the mortality rate is increasing between 24 and 48h whilst control mortality remains at an accepted level, i.e. <10%, it is appropriate to extend the duration of the test to a maximum of 96 h. Mortality is recorded daily and compared with control values. The results are analysed in order to calculate the LD_{50} at 24, 48h, 72h, and 96h (see Annex for definitions).

Note that for this ring test the full test duration of 96h is required.

VALIDITY OF THE TEST

7. For a test to be valid the following conditions apply:
   - The average mortality for the total number of controls must not exceed 10 % at the end of the test.
   - The LD_{50} of the toxic standard Dimethoate 40% meets the specified range. As solitary bees differ more in size / weight than honeybees, a larger variation in LD_{50} values can be observed. For *Osmia* the LD_{50} of Dimethoate approximates 1.5 µg a.i./bee. For other solitary bee species the LD_{50} may be significantly different.

Note that depending on the results of the ring test the control mortality criterion might be changed to 15 to 20% in accordance to other non-target arthropod testing.

DESCRIPTION OF THE METHOD

Collection of bees

8. Newly emerged female bees (preferably of modal size) are selected for the test. Cocoons containing females are generally of larger size than those containing males. Do not manipulate the cocoons in order to facilitate hatching and/or sexing of the bees (i.e. do not open or cut prior to hatching). When hatching proper sized cocoons in a flight cage, any males still present will emerge earlier than the females and should be removed from the cage. Emerged females should be non-mated and meconium-free and are to be stored in the refrigerator at 5 ºC until enough bees have been collected to populate the test. Note that this can take up to 4 days since 30 bees per treatment group are required.

Number of bees per treatment group

9. Thirty (30) non-mated meconium-free solitary bee females.

Number of doses

10. Per test the bees are treated with 5 doses of the test substance: two between the presumed LD_{100} and LD_{50}, one at the presumed LD50 and two between the presumed LD_{50} and LD_0, a negative control (in case a solvent is used) and at least three concentrations of the positive control.

Note that in the current ring test, dimethoate (positive control substance when testing other chemicals) is tested so no positive control is required in the current ring test.

Number of replicates

11. An acute contact LD_{50} consists of three [5] replicates in parallel to be executed as 3 x 10 bees from the same geographic pool/supplier. However, good results have also been obtained by participants using 6x5 bees. Both designs are considered adequate. At all times the origin, normal flight period in the year and wintering conditions of the cocoons of the bees used in the test must be specified in the raw data.

Note that in the current test only group housing i.e. 3x10 or 6x5 bees per replicate will be tested.

Test cages

12. Easy to clean and well-ventilated cages are used. Any appropriate material can be used, e.g. stainless steel, wire mesh, plastic, disposable plastic cages, et cetera. The size of test cages should be appropriate to the number of bees, i.e. providing adequate space and feeding opportunity (i.e. all individuals should have access to the sugar solution). This can be arranged by using bigger cages with multiple feeders or using less bees per cage, but increasing the number of cages per treatment level. In principle, however, groups of 10 bees per cage are tested. Provide cage enrichment like a piece of gauze and/or (filter)paper, since *Osmia* bees like to play around/have hiding places. Food should be available *ad libitum* and feeders should be placed on the ground of the test cage (*Figure 1*).

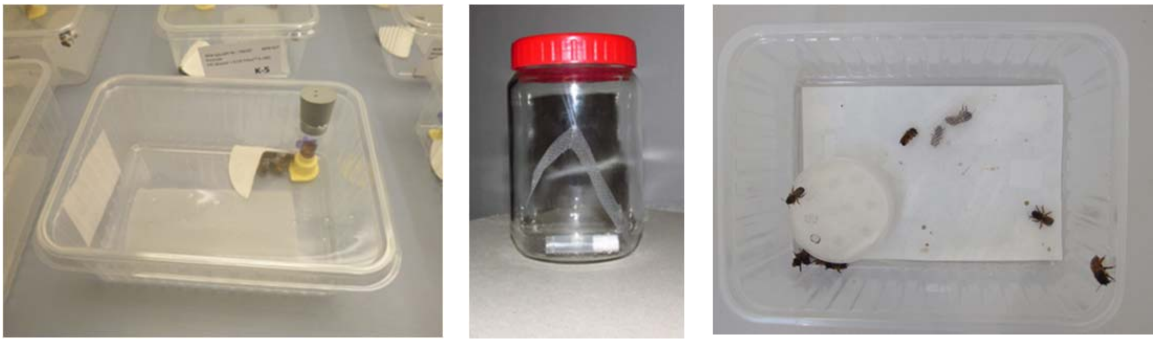

Figure 1. Some examples of test cages with feeders positioned on the ground.

Note that feeders positioned on the ground appear to work better than suspended feeders. Hence in the ring test, feeders need to be positioned on the ground. Feeders containing a reservoir with some kind of wick or cotton from which the Osmia feed give good results, but good results are also obtained with feeders equipped with a flower petal. Participants are asked to fine-tune their choice feeders with the coordinator before testing so that a balanced ring test using both feeder types can be performed.

Preparations of bees

13. The collected bees are anaesthetized by chilling by putting them for at least 30 minutes at 4-5 °C or using an ice bath. Cold storage can be prolonged but the amount/duration of anaesthetic used and times of exposure should be minimised. Note that using CO_2 for anaesthesia can result in mortality for *Osmia* species and should therefore be avoided. All bees are weighed before application of the test substance to determine the average weight, standard deviation and min-max weight of animals used in the test. Moribund bees should be rejected and replaced by healthy bees before starting the test.

Preparation of doses

14. All test item doses will be dissolved in water. Add Triton X-100 (0.1%) as surfactant or any other low toxic surfactant which equally distributes the droplet on the bee body.

Note that in this ring test Triton X should be used as a surfactant. When testing formulated products, the test substance is dissolved in water.

Housing and keeping of the solitary bees

15. The bees are kept under light: dark conditions (16:8h) in a climate room at a temperature of 22 ± 2°C and a relative humidity of 60 ±10%. During the test the bees have access to sucrose solution 50% (w/w) ad libitum.
16. Per test cage ten bees will be housed (3x10). If using the 6x5 option, five bees per test cage will be used.

Handling and feeding conditions

17. Handling procedures, including treatment and observations may be conducted under (day)light.

Test item ring test

18. The ring test will be performed using Dimethoate 40% (e.g. Dimethoate 400 EC).

Test concentrations ring test

19. The proposed test-range for the ring test is: control, 0.5, 1.0, 2.0, 4.0, 8.0 µg active ingredient / bee

Note that this range has been slightly adapted, compared to the range used in 2015.

Administration of doses

20. Anaesthetized bees are individually treated by topical application. The bees are randomly assigned to the different test substance doses and controls. A volume of 2 µL of solution containing the test substance at the suitable dose should be applied with a micro-applicator to the dorsal side of the thorax of each bee between the neck and wing base. After application, the bees are allocated to test cages in groups of 10 bees and supplied with sucrose solutions 50% ad libitum.

Residue analyses test substance

21. At minimum, the stock solution, the lowest, and the highest test concentrations are analysed for Dimethoate levels. Till analysis, the solution of the test substance is stored in the freezer (-18 ºC).

PROCEDURE

Test and control groups

22. The number of doses and replicates tested should meet the statistical requirements for determination of LD_{50} with 95% confidence limits. Normally, five doses in a geometric series, with a factor not exceeding 2.2, and covering the range for LD_{50}, are required for the test. However, the number of doses has to be determined in relation to the slope of the toxicity curve (dose versus mortality) and with consideration taken to the statistical method which is chosen for analysis of the results. A range-finding test enables the choice of the appropriate doses (not applicable for the current dimethoate ring test).

Note that participants are asked to send in their raw data in the distributed format so that all data can be processed in an uniform manner.

23. A minimum of three replicate test groups, each of 10 bees, should be dosed with each test concentration (not applicable for the current dimethoate ring test).

24. A minimum of three replicate cages, each containing 10 bees, should be used with each test dose. Note that when 6 replicates are used, each can contain 5 bees.

Exposure

Test conditions

25. The bees should be held under light: dark conditions (16:8h) in a climate room at a temperature of 22 ± 2°C and a relative humidity of 60 ±10%. During the test the bees have access to sucrose solution 50% (w/w) ad libitum.

Duration

26. The duration of the test is 96 h.

Observations

27. Mortality is recorded at 4 h after dosing and thereafter at 24h, 48 h, 72 h, and 96 h. All abnormal behavioural effects observed during the testing period should be recorded.

DATA AND REPORTING

Data

28. Data should be summarised in tabular form, showing for each treatment group, as well as control and toxic standard groups, the number of bees used, mortality at each observation time and number of bees with adverse behaviour. Analyse the mortality data by appropriate statistical methods (e.g. probit analysis, moving-average, binomial probability) [5, 6]. Plot dose-response curves at each recommended observation time (i.e. 24h, 48h, 72h, and 96h) and calculate the slopes of the curves and the median lethal doses (LD_{50}) with 95% confidence limits. Corrections for control mortality could be made using Abbott´s correction or Scheider Orelli [7, 8]. LD_{50} should be expressed in µg of test substance per bee and µg of test substance per gram bee.

Note that participants are asked to send in their raw data in the distributed format so that all data can be processed in an uniform manner.

Test report

29. The test report must include the following information:

Test substance:
- physical nature and relevant physical-chemical properties (e.g. stability in water, vapour pressure);
- chemical identification data, including structural formula, purity (i.e. for pesticides, the identity and concentration of active ingredient (s)).

Test bees:
- scientific name, race, approximate age (in weeks), collection method, date of collection;
- all relevant information on colonies used for collection of test bees, including health, any adult disease, any pre-treatment, etc.

Test conditions:
- temperature and relative humidity of experimental room;
- housing conditions including type, size and material of cages;
- methods of administration of test substance, e.g. carrier solvent used, volume of test solution applied, anaesthetics used;
- test design, e.g. number and test doses used, number of controls; for each test dose and control, number of replicate cages and number of bees per cage;
- date of test.

Results:
- results of preliminary range-finding study if performed;
- raw data: mortality at each concentration tested at each observation time;
- graph of the dose-response curves at the end of the test;
- LD_{50} values, with 95% confidence limits, at each recommended observation time, for test substance and toxic standard;
- statistical procedures used for determining LD_{50};
- mortality in controls;
- other biological effects observed and any abnormal responses of the bees;
- any deviation from the Test Guideline procedures and any other relevant information.

Note that participants are asked to send in their raw data in the distributed format so that all data can be processed in an uniform manner.

LITERATURE

1. OECD, *Honeybees - acute oral toxicity test*, in *OECD Guidelines for the testing of chemicals No. 213*1998, Organization of Economic Co-operation and Development: Paris.
2. Steen. J.J.M. van der, C. Gretenkord, and H. Schaefer. *Methods to determine the acute oral and contact LD50 of pesticides for bumble bees (Bombus terrestris L.)* in *Proceedings ICPBR 6th Symposium on the Hazard of Pesticides to Bees* 1996. Braunschweig, Germany.
3. Johansen, C.A., et al., *Pesticides and Bees.* Environmental Entomology, 1983. **12**(5): p. 1513-1518.
4. Steen, J.J.M.v.d. *The effect of the size of the solitary bee (Bombus terrestris L.) on the susceptibility to the pesticide dimethoate 40%* in *Proceedings 7th International Symposium of the ICPBR Bee Protection Group co-organised by INRA and ACTA 1999:213-216. INRA Editions RD 10-78026 Versailles Cedex, France. ISBN 2-7380-0966-2.* 2001.
5. Finney, D.J., *Probit Analysis. 3rd ed., Cambridge, London and New York.* 1971, Cambridge, London and New York. 333.
6. Litchfield, J.T. and F. Wilcoxon, *A simplified method of evaluating dose-effect experiments.* Journal of Pharmacology and Experimental Therapeutics, 1949. **96**(2): p. 99-113.
7. Schneider - Orelli, O., *Entomologisches Praktikum : Einfuehrung in die land- und forstwirtschaftliche Insektenkunde*. 1947, Aarau: Sauerlaender.
8. Abbott, W.S., *A method for computing the effectiveness of an insecticide.* Jour. Econ. Entomol., 1925. **18**: p. 265-267.

ANNEX

DEFINITIONS

Acute contact toxicity is the adverse effects occurring within a maximum period of 96 h of a topical application of a single dose of a substance.

Dose is the amount of test substance applied. Dose is expressed as mass (µg) of test substance per test animal (µg/bee).

LD_{50} (median lethal dose) contact, is a statistically derived single dose of a substance that can cause death in 50 per cent of animals when administered by contact. The LD_{50} value is given in µg of test substance per bee and per gram bee. For pesticides, the test substance may be either an active ingredient (a.i.) or a formulated product containing one or more than one active ingredient.

Mortality: an animal is recorded as dead when it is completely immobile.

Additionally to the LD_{50} the test duration time in which the LD_{50} is calculated, 24, 48, 72 or 96 h is presented.
