# Supplementary material for: Is Osmia bicornis an adequate regulatory surrogate? Comparing its acute contact sensitivity to Apis mellifera
Source: PLoS One. 2019 Aug 8;14(8):e0201081. doi: 10.1371/journal.pone.0201081 (PMC6687126; doi:10.1371/journal.pone.0201081)
Supplement: S1 Table — For a detailed account of raw data from all tests see Uhl et al. (2018) [26]. (PDF) [file pone.0201081.s003.pdf]

Table S1: Overview of all tested insecticides and test dates. For a detailed account of raw data from all tests see Uhl et al. (2018).

Study code	Insecticide (a.i.)	Product	Application date
Ro_Ob_ALPHA_1	alpha-cypermethrin	FASTAC® SC	2016-07-08
OA_Ob_BCYF	beta-cyfluthrin	Bulldock®	2017-07-30
OA_Ob_DEL	deltamethrin	Decis® Forte	2017-07-30
Ro_Ob_ETOFEN_1	etofenprox	Trebon® 30 EC	2016-07-08
Ro_Ob_LAMBDA_1	lambda-cyhalothrin	Karate® Zeon	2016-05-28
OA_Ob_ZCYP	zeta-cypermethrin	Fury® 10 EW	2017-07-30
OA_Ob_ACE	acetamiprid	Mospilan® SG	2017-08-06
Ro_Ob_IMI_1	imidacloprid	Confidor® WG 70	2016-05-28
Ro_Ob_THIA_1	thiacloprid	Calypso®	2016-08-03
CW_Ob_DIM	dimethoate	PERFEKTHION®	2015-05-21
OA_Ob_DIM	dimethoate	PERFEKTHION®	2017-06-08
OA_Ob_CHL	chlorpyrifos	Pyrinex®	2017-08-06
Ro_Ob_CHLORAN_1	chlorantraniliprole	Coragen®	2016-08-04
Ro_Ob_FLUPY_1	flupyradifurone	Sivanto® SL 200 G	2016-08-04
OA_Ob_IND	indoxacarb	AVAUNT® 150 EC	2017-06-29
Ro_Ob_PIRI_1	pirimicarb	Pirimor®	2016-07-08
Ro_Ob_SPINO_2	spinosad	SpinTor®	2016-08-03

References

Uhl, P., Awanbor, O., Schulz, R. S., & Brühl, C. A. (2018). Raw data - Ecotoxicological tests with Osmia bicornis and 16 insecticides. *figshare. Fileset*, doi:10.6084/m9.figshare.6143945.v9. Available from: https://figshare.com/articles/Raw_data_-_Ecotox_tests_with_16_insecticides/6143945/9
