# Supplementary material for: Is Osmia bicornis an adequate regulatory surrogate? Comparing its acute contact sensitivity to Apis mellifera
Source: PLoS One. 2019 Aug 8;14(8):e0201081. doi: 10.1371/journal.pone.0201081 (PMC6687126; doi:10.1371/journal.pone.0201081)
Supplement: S2 Table — (PDF) [file pone.0201081.s004.pdf]

Table S2: Data sources of honey bee acute endpoints for all tested insecticides.

| Insecticide (a.i.) | Product | Data source | Comment |
|---|---|---|---|
| acetamiprid | Mospilan® SG | RAR (2015) | |
| alpha-cypermethrin | FASTAC® SC | personal communication UBA | |
| beta-cyfluthrin | Bulldock® | RAR (2017) | modelled from 48 h mean mortality data |
| chlorantraniliprole | Coragen® | DAR (2008) | |
| chlorpyrifos | Pyrinex® | RAR (2017) | |
| deltamethrin | Decis® Forte | personal communication UBA | validated by Mark Miles (Bayer Crop Science) |
| dimethoate | PERFEKTHION® | RAR (2017) | |
| etofenprox | Trebon® 30 EC | NA | |
| flupyradifurone | Sivanto® SL 200 G | DAR (2014) | |
| lambda-cyhalothrin | Karate® Zeon | RAR (2013) | |
| imidacloprid | Confidor® WG 70 | DAR (2005) | |
| indoxacarb | AVAUNT® 150 EC | RAR (2016) | |
| pirimicarb | Pirimor® | RAR (2017) | comparable value to Pirimor® according to RAR |
| spinosad | SpinTor® | RAR (2017) | |

Table S2: Data sources of honey bee acute endpoints for all tested insecticides (continued).

| Insecticide (a.i.) | Product | Data source | Comment |
|---|---|---|---|
| thiacloprid | Calypso® | EC review report (2004) | validated by Mark Miles (Bayer Crop Science) |
| zeta-cypermethrin | Fury® 10 EW | DAR (2006) | |
