# Supplementary material for: Is Osmia bicornis an adequate regulatory surrogate? Comparing its acute contact sensitivity to Apis mellifera
Source: PLoS One. 2019 Aug 8;14(8):e0201081. doi: 10.1371/journal.pone.0201081 (PMC6687126; doi:10.1371/journal.pone.0201081)
Supplement: S3 Table — (PDF) [file pone.0201081.s005.pdf]

Table S3: Different organisations that aided with data collection and contact at the respective institutions.

Organisation	Contact
German Environment Agency (UBA)	Dirk Süßenbach
German Federal Office of Consumer Protection and Food Safety (BVL)	Rolf Forster
European Food Safety Authority (EFSA)	Csaba Szentes
Bayer Crop Science	Mark Miles
Dow AgroSciences	Anne Alix
Syngenta	Robert Spatz
