# Supplementary material for: Is Osmia bicornis an adequate regulatory surrogate? Comparing its acute contact sensitivity to Apis mellifera
Source: PLoS One. 2019 Aug 8;14(8):e0201081. doi: 10.1371/journal.pone.0201081 (PMC6687126; doi:10.1371/journal.pone.0201081)
Supplement: S1 Fig — (PDF) [file pone.0201081.s006.pdf]

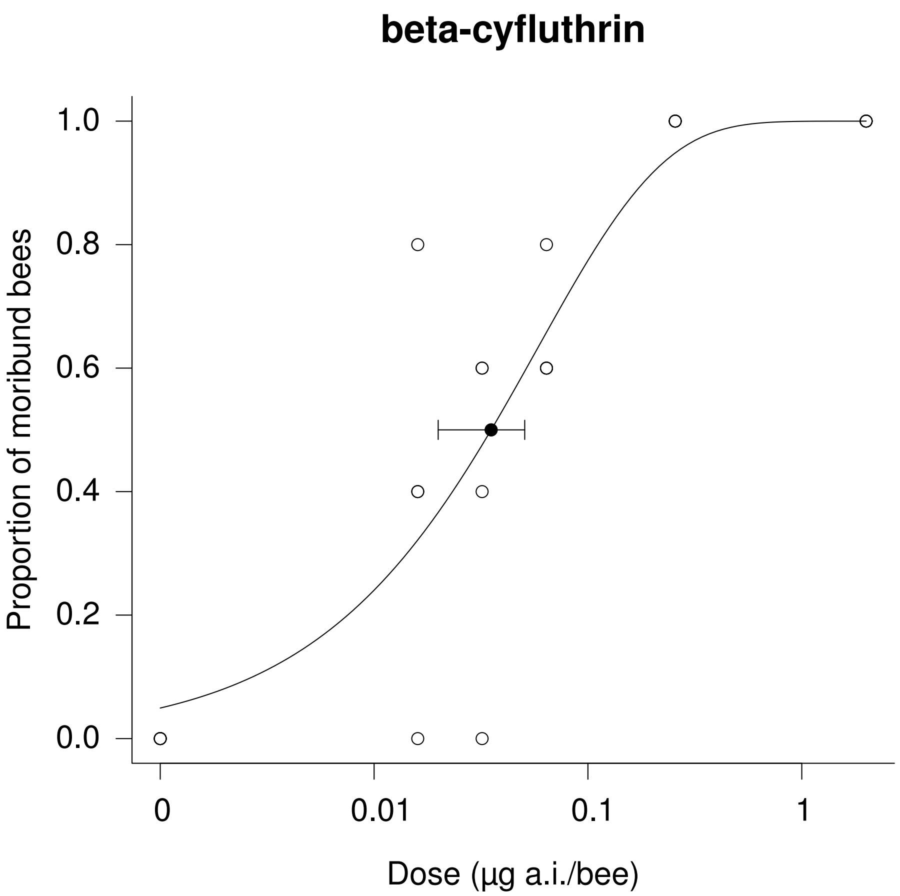

Figure S1: Dose-response curve from *O. bicornis* 48h contact toxicity test with beta-cyfluthrin. Study code: OA_Ob_BCYF.
