# Supplementary material for: Is Osmia bicornis an adequate regulatory surrogate? Comparing its acute contact sensitivity to Apis mellifera
Source: PLoS One. 2019 Aug 8;14(8):e0201081. doi: 10.1371/journal.pone.0201081 (PMC6687126; doi:10.1371/journal.pone.0201081)
Supplement: S12 Fig — (PDF) [file pone.0201081.s017.pdf]

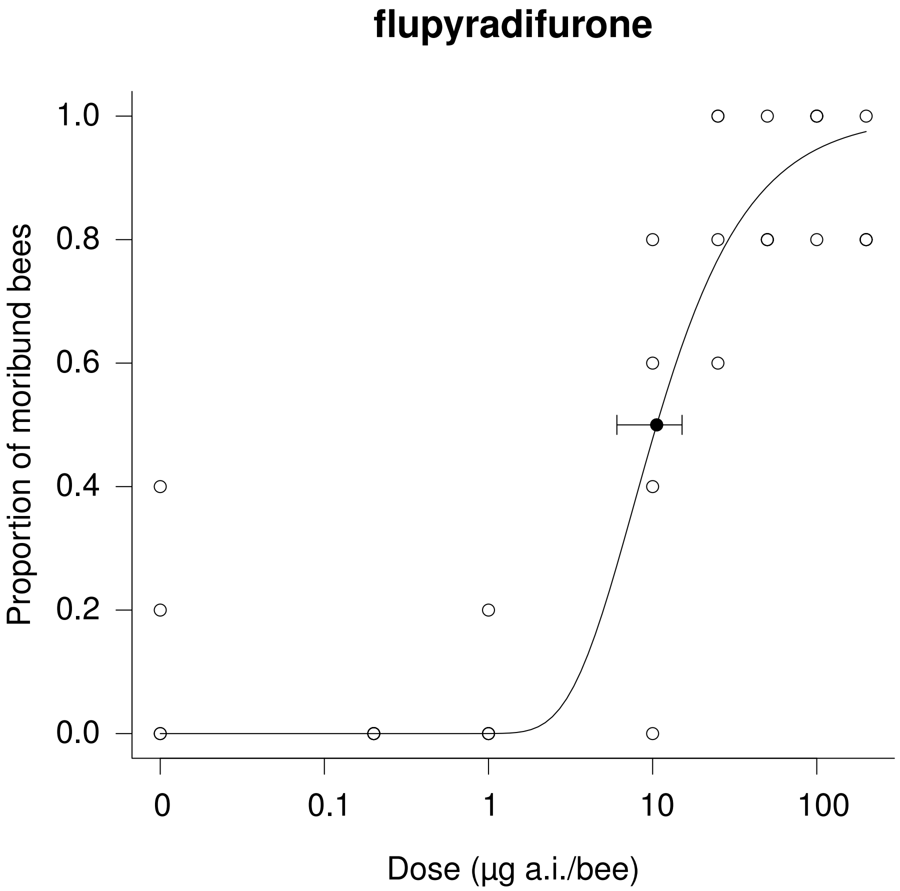

Figure S12: Dose-response curve from *O. bicornis* 48h contact toxicity test with flupyradifurone. Study code: Ro_Ob_FLUPY_1.
