# Supplementary material for: Is Osmia bicornis an adequate regulatory surrogate? Comparing its acute contact sensitivity to Apis mellifera
Source: PLoS One. 2019 Aug 8;14(8):e0201081. doi: 10.1371/journal.pone.0201081 (PMC6687126; doi:10.1371/journal.pone.0201081)
Supplement: S16 Fig — (PDF) [file pone.0201081.s021.pdf]

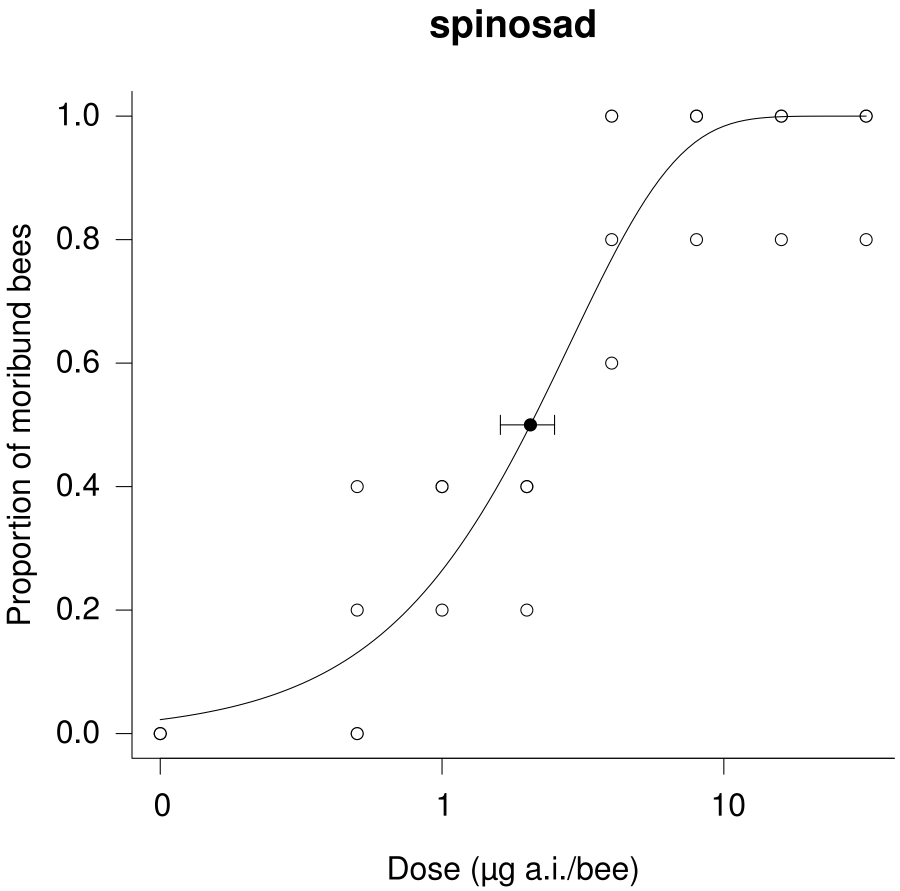

Figure S16: Dose-response curve from *O. bicornis* 48h contact toxicity test with spinosad. Study code: Ro_Ob_SPINO_2.
